# Natural Preservatives from Plant in Cheese Making

**DOI:** 10.3390/ani10040749

**Published:** 2020-04-24

**Authors:** Mena Ritota, Pamela Manzi

**Affiliations:** CREA—Centro di Ricerca Alimenti e Nutrizione, Via Ardeatina 546, 00178 Rome, Italy; pamela.manzi@crea.gov.it

**Keywords:** natural preservatives, cheese making, antimicrobial, functional properties, sensory characteristics, shelf-life

## Abstract

**Simple Summary:**

Cheese is a food that is susceptible to contamination by pathogenic and spoilage microorganisms, which can result in a reduced cheese shelf life, as well as in risks to the consumers’ health. This implies the possible use of preservatives in the cheese making process. At the same time, consumers are increasingly demanding for healthy food, free from synthetic preservatives. Just for this reason, natural ingredients are receiving increasing attention as substitutes for synthetic additives, also because they have bioactive compounds, which might provide health benefits in the prevention of several diseases. Furthermore, most of natural ingredients have shown antimicrobial activity, which could delay or inhibit the growth of pathogenic microorganisms in food, as well as minimize the incidence of foodborne diseases caused by food spoilage bacteria and fungi. This review aims at discussing the antimicrobial activity of the main natural ingredients derived from plants and used in cheese making, and their effect on cheese quality, in terms of chemical, nutritional and sensory characteristics of the products, as well as the increase in cheese shelf-life.

**Abstract:**

Today, consumers are increasingly demanding safety alternatives concerning the use of synthetic additives in the food industry, as well as healthy food. As a result, a major number of plant-derived preservatives have been tested in the food industry. These natural ingredients have antioxidant properties and have shown to increase the bioactive molecules levels and the microbiological stability of the food items. The effect of the plant-based preservatives on the sensorial properties of the new products has also to be considered, because natural preservatives could result in sensorial characteristics that may not be accepted by the consumers. Cheese is a dairy product widely appreciated all over the world, but it is also susceptible to contamination by pathogenic and spoilage microorganisms; therefore, the use of preservatives in cheese making represents an important step. This review deals with one of the innovation in the cheese sector, which is the addition of natural preservatives. Several aspects are discussed, such as the effect of natural ingredients on the microbial stability of cheese, and their influence on the chemical, nutritional and sensorial characteristics of the cheeses. Although the promising results, further studies are needed to confirm the use of natural preservatives from plants in cheese making.

## 1. Introduction

Following the rapid globalization of food production and trade, there has been a marked increase in the likelihood of international incidents involving contaminated food: just remember the melamine incident in China in 2008 [1], the German *Escherichia coli* O104:H4 outbreak in 2011 [2], and others.

The urgent need to address food safety on a global scale led to the creation in 2004 of a joint program of the Food and Agriculture Organization of the United Nations (FAO) and the World Health Organization (WHO), the International Food Safety Authorities Network (INFOSAN) [3], aiming at preventing the international spread of contaminated food and foodborne disease and strengthening the food safety systems globally.

European Union (EU) was among the first to adopt regulation regarding food hygiene and safety [4,5,6]. The overall objective of these hygiene rules is to guarantee a high level of consumer’s protection with regard to food safety, by adopting an integrated approach, in which each food chain operator must ensure that food safety is not compromised along the entire food chain.

A significant problem affecting almost the entire food chain is the high risk to be contaminated by pathogenic and spoilage microorganisms. Dairy products, in particular, are generally susceptible to contamination: in the dairy industry, and in particular at small farm production, multiple sources of contamination have proven to be the use of raw milk, the dairy environment, and in some cases also vegetable coagulants [7,8]. Yeasts and molds play an important role in the spoilage of dairy products. Mold spoilage is generally promoted by a humidity excess in the ripening environment: if mold remains outside the wheel, it is almost never harmful and can be easily removed by periodic washing; however, if mold goes through the wheel, it may cause defects in the cheese, such as off-flavors [9] and changes in the texture and color. Furthermore, the growth of mold in cheese could result in serious consequences on consumers’ health, due to the molds production, in particular conditions, of secondary metabolites, generally referred to as mycotoxins, highly toxic compounds, which, if ingested in large quantities, can induce hepatitis, hemorrhages, and necrosis, up to death [10,11]. Although many cheeses undergo a heat treatment during the cheese making process, cheese is generally susceptible to contamination by microorganisms, which can result in cheese spoilage, health risks for consumers, and reduction in the cheese shelf life. Therefore, the cheese rind treatment with preservatives becomes a necessary step in cheese making, especially during long ripening times.

According to the Regulation of European Community (EC) No. 1333/2008 [12] on food additives, preservatives are defined as “substances which prolong the shelf-life of foods by protecting them against deterioration caused by micro-organisms and/or which protect against growth of pathogenic micro-organisms”. The Commission Regulation (EU) No. 1129/2011, amending Annex II to Regulation (EC) No 1333/2008 [12], establishes a Union list of food additives, including the use of preservatives such as sorbic acid and sorbates (E 200–203) for unripened and ripened cheese, nisin (E 234) only for mascarpone and ripened cheeses, and acetic acid (E 260) and lactic acid (E 270) only for mozzarella [13]. The use of lysozyme (E 1105), propionic acid and propionates (E 280–283), and in some cases also natamycin (E 235), hexamethylene tetramine (E 239), and nitrates (E E251–252) is allowed for ripened cheese [13]. Although these preservatives are safe for human health in the allowed dosage, but they are extensively used in the food industry, the consumption of a large amount of these additives may give rise to certain health problems [14]. Therefore, concerns about the safety of some chemical preservatives and the negative consumers’ perception towards synthetic additives have led to a growing interest in more natural alternatives, among which plant-based compounds [15].

Recently, many scientific works [16,17] reviewed the latest developments regarding the use and effects of herbal extracts in the dairy sector, showing how a large number of natural compounds can be added into different formulations. The use of herbs and spices in cheese making is a widespread practice, since ancient times, but it generally involved physically rubbing the cheese with certain herbs or spices, or their oils [18], and is often related to local traditions. There are numerous cheeses treated with natural herbs, in particular in Italy (*Casoperuto, Marzolino, Romano pepato, Piacentinu Ennese*, etc.), where the dairy tradition is very old, but also in many other European countries, such as Switzerland (*Swissalp Panorama, Bellevue*), France (*Le Roule, Boulette d’Avesnes*), Netherland (*Kanterkass*), as well as in many other countries in the world, such as Egypt (*Karish*), Syria (*Shankalish*), Morocco (*Jben* and *Raib*), and Turkey (*Otlu*, *Surk*, and *Carra*). However, the main concern regarding the use of different species of native aromatic herbs in cheese making could be the high microbial load on the surface of plant leaves [19]. Therefore, scientific research is currently focused on the development of new methods for using natural extracts as preservatives [20]. When referring to plant extracts, they obviously should obtained by using no toxic organic solvents, such as water, ethanol, or their binary mixtures [17], and the extracts should derive from herbs with recognized and traditional uses [21].

**Figure 1:**
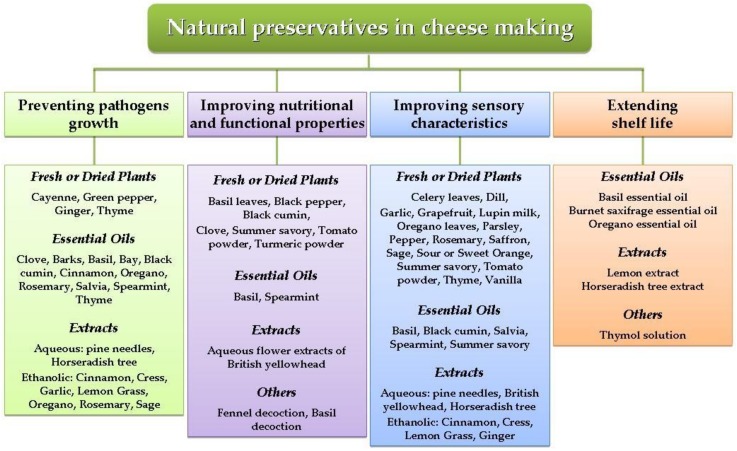
Major plants and their applications in cheese making.

This review aims at giving an overview of the recent applications of plant-based compounds in the dairy sector. Even if it does not deal with natural antimicrobials from bacterial (bacteriocins) or animal origin (lactoferrin, chitosan, lysozyme, etc.), the paper wants to focus on the role of natural compounds from plants in cheese for reducing or eliminating pathogenic and spoilage bacteria, and increasing the overall quality of cheese, focusing, above all, on the last decade literature. A summary of all applications of plant-based compounds in cheese making discussed in this review is reported in Figure 1.

## 2. Use of Natural Preservatives in Preventing Pathogens Growth in Cheese

Antimicrobials are generally used for food preservation, by controlling natural spoilage processes, and for food safety, by preventing/controlling the microorganisms growth, including pathogenic microorganisms [22].

Natural compounds exerting antimicrobial activity, and found in higher concentrations into the plants, are phenolic compounds, as well as terpenoids, sesquiterpenes, and possibly diterpenes with different groups [22]. Antimicrobial activity of *Thymus algeriensis* essential oil (EO) is, for instance, ascribed to its major monoterpenoid phenol compound, carvacrol [23], while benzene derivatives seem to play a major role in terms of antimicrobial activity in the essential oil of various species of *Pimpinella anisum* L. plant [24]. The antimicrobial as well as the health related effects in *Allium* species are, instead, attributed to the major sulphur containing compounds found in the plants, in particular diallyl sulphides [25], while the antimicrobial activity of spearmint essential oil is related to its major terpenoid compounds, carvone and limonene [26]. Finally, eugenol and thymol are the most important representatives with antibacterial and antifungal activities from clove and thyme oils, respectively [18], while antimicrobial activity of ginger is related to several compounds such as gingerol, gingerdiol, and shogaol [27].

Natural substances with antimicrobial activity seem to act on the permeabilization or disruption of the cytoplasmic membrane, thus allowing, respectively, the passage or the release of nonspecific compounds. Furthermore, they may inhibit the key enzyme of the cell energy generation (ATPase), thus leading to the cell death [28].

The antimicrobial activity of the plant-derived compounds in cheese can be carried out both in terms of antibacterial and antifungal activities.

### 2.1. Use of Natural Preservatives as Antibacterials in Cheese

The antibacterial activity of natural compounds is generally evaluated against the major pathogenic microorganisms commonly reported in cheeses, such as *Listeria monocytogenes*, *Staphylococcus aureus*, *Escherichia coli*, and *Salmonella* spp. [28]. 

Among the most effective plants, black cumin seed oil supplemented to a soft cheese showed a general antibacterial activity against all main cheese pathogenic bacteria [29]. Cayenne and green pepper were able to reduce *S. aureus* population in Egyptian *Kareish* cheese [30], while extracts of cinnamon, garlic, lemon grass, cress, rosemary, sage, and oregano individually inhibited the population of *L. monocytogenes* in processed cheeses [31]. Furthermore, different spices have shown dissimilar behavior against various pathogenic microorganisms [32].

Extensive research has been focused on the use of essential oils of aromatic plants in food preservation, their antimicrobial activities being widely recognized [33], even if Gram-negative bacteria are partly more resistant to antimicrobial essential oils, due to the existence of lipopolysaccharide in their outer membranes [34,35]. Oregano and thyme essential oils have shown to exert antimicrobial activity against *L. monocytogenes* in feta cheese [36], while in Iranian white cheese, salvia, and basil essential oils showed antimicrobial activity against *L. monocytogenes* at concentrations <0.1% and <1%, respectively [37].

The antibacterial effects of black cumin essential oil against *E. coli* O157:H7 and *L. monocytogenes* were evaluated in samples of Iranian white cheese inoculated with these pathogens [38]. In the cheeses treated with black cumin EO the growth of both pathogens was significantly lower compared to the control during storage, in particular for *L. monocytogenes*, confirming that Gram-negative bacteria are generally less sensitive than Gram-positive bacteria to the antibacterial effect of essential oils [34,35].

Recently, also aqueous extracts have been evaluated as potential natural preservatives. Mahajan et al. [39], for example, reported that aqueous extracts of pine needles (*Cedrus deodara* (Roxb.) Loud.) improved the lipid oxidative stability of low fat *Kalari*, a typical Indian hard and dry cheese, as well as its microbiological characteristics, due to the antioxidant and antimicrobial properties of the pine needles phytochemicals. 

### 2.2. Use of Natural Preservatives as Antifungals in Cheese

Some plant-based compounds have also shown promising results in inhibiting the growth of pathogenic fungi [28]. Fungi are significant spoilage microorganisms of foodstuffs during storage, resulting in foods unfit for human consumption, by reducing their nutritive value and sometimes by producing mycotoxins [40]. Common cheese contaminants are *Penicillium* and *Aspergillus* [41]. Wendorff et al. [42] showed how it was possible to reduce fungal growth on the surface of the cheese through the use of liquid smoke, while olive oil, used as a surface treatment during cheese ripening, has revealed to increase or decrease the growth of molds, depending on whether the rind is already formed or not at the moment of treatment [43]. Quinto et al. [44] evaluated the effect of different surface treatments (olive oil, liquid smoke, and pimaricin) on the ripening of the *Canestrato Pugliese*, highlighting how the development of molds, together with the proteolytic and lipolytic activity of the surface layer, are strongly influenced from the rind treatments during ripening. In particular, the treatment with olive oil showed the highest number of counts and the largest number of species identified, confirming the hypothesis of Wendorff and Wee [43]. Jeong et al. [45] evaluated the in vitro antifungal activity against *Penicillium* spp. of different essential oils (cinnamon leaf or barks, basil, ginger, lemon, peppermint, pine needle, and spearmint): cinnamon leaf and barks essential oils showed the highest antifungal activity and were tested as antimicrobials during Appenzeller cheese ripening. The authors reported an optimal concentrations of cinnamon essential oils ≤10% (*v*/*v*) to both gain antimicrobial activity and not inhibit the growth of the cheese starters [45].

Suárez et al. [46] showed that also polyphosphate-based treatments inhibited the superficial development of molds in hard cheeses. Finally, Balaguer et al. [47] showed that incorporation of cinnamon essential oil, containing 5% cinnamaldehyde, into a film coating a spreadable cheese delayed the growth of *Apergillus niger* and *Penicillium expansum*.

### 2.3. Relevant Aspects Concerning the Use of Natural Preservatives in Cheese Making

One of the main concerns regarding the use of plant-based compounds as natural preservatives is about their effective potential in inhibiting the natural microbial population or artificial starter microorganisms added to cheese [28]. Marcial et al. [48], when evaluating the influence of oregano essential oil (EO) on a traditional Argentinean cheese, reported that oregano EO had no effect on the growth and acidifying activity of lactic acid bacteria (LAB) in milk, besides improving the microbiological quality of the products during ripening. Additionally rosemary essential oil had no inhibitory effect on the lactic flora of a sheep milk cheese, while simultaneously prevented the growth of *Clostridium* spp., responsible for the late cheese blowing [49]. In the same way, Gammariello et al. [50] confirmed that the growth of lactic acid bacteria in *Fior di latte, a pasta filata* cheese, was not affected by the presence of natural compounds with antimicrobial activity. Mohamed et al. [51] evaluated the effect of different extracts of *Moringa oleifera* leaves on the growth of probiotic bacteria in a cream cheese: both ethanolic and aqueous extracts did not inhibit the LAB growth, but ethanolic extract resulted in a higher growth. Licón et al. [52], instead, reported that essential oil of *Melissa officinalis,* at a concentration of 250 mg/kg, was not suitable as antimicrobial in pressed ewes’ cheese, because it showed inhibitory effect against lactic acid bacteria starter cultures, unlike essential oils of *Ocimum basilicum* and *Thymus vulgaris*.

Another important aspect (often overlooked by the scientific literature) when considering natural antimicrobial agents is the food matrix influence. Smith-Palmer et al. [53], in fact, observed that the cheese composition was a significant factor in determining the antimicrobial activity of some natural compounds, since only in low-fat quark type cheese all the essential oils tested (bay, clove, cinnamon, and thyme) where effective in inhibiting the growth of *L. monocytogenes*, while in high fat cheese only clove essential oil showed the same activity. Similarly, Gutierrez et al. [54] reported that the antimicrobial activities of essential oils of oregano and thyme against *Listeria monocytogenes* were reduced by high lipid concentrations in a media simulating a food matrix.

Furthermore, if the inhibitory capacity of a specific concentration of a natural compound has been previously tested in laboratory, the same natural compound should most likely be added in food in larger quantities than the level tested in vitro to provide the same inhibitory effect [55]. Natural compounds, in fact, can be lost during cheese making, due to their solubility in the whey [49] or to their sensibility to light, temperature, oxygen, and pH [56]. This was also confirmed by the results of Gammariello et al. [50], who observed that a higher concentration of the natural compounds was needed to achieve the same antimicrobial effect in Fior di latte cheese, than that tested in vitro. In the same way, Da Silva Dannenberg et al. [57] observed that the concentration of essential oil from pink pepper tree that corresponded to the minimum inhibitory concentration against *L. monocytogenes* in an in vitro assay was not effective in the Minas-type fresh cheese in controlling the same pathogen. An alternative approach to reduce the in vivo concentration of these plant-derived ingredients is to use a combination of them: some compounds, in fact, showed higher antimicrobial effect when using in the mixture than when used alone [54], resulting in a synergistic effect. Moreover, microencapsulation could also be an emerging technology to ensure better stability of these compounds during cheese making [28], while preserving their antioxidant activity along the products shelf life [58].

A further aspect concerning the use of natural antimicrobials, both plant extracts and essential oils, is the difficult in defining the specific quantity of the natural compounds to be added to food, in order to ensure the expected antimicrobial effect [28]. Concentration of the plants bioactive compounds exerting antimicrobial activity, in fact, are determined by plants genetics, and may change due to different factors, such as soil composition, climate, plant management, and phenological stage: all these variables can represent a limitation to the use of plant extracts and essential oils as food preservatives [28]. According to the data reported in the literature, the concentrations of natural compounds in food should range from 0.05% to 0.1% (500–1000 ppm) to be effective [22]. However, some scientific works reported higher value, such as Vrinda Menon et al. [59], who observed inhibitory effect of clove oil on *Listeria monocytogenes* in cheese at a concentration ranging between 0.5% and 1%, with pronounced antimicrobial activity at 1% clove oil. Similarly, Selim [60] observed antimicrobial activity of various essential oils against Vancomycin-resistant *Enterococci* and *Escherichia coli* O157:H7 in feta soft cheese in a concentration between 0.1% and 1%, with thyme EO being the most active.

Furthermore, it is worthwhile highlighting that the extracting solvent has revealed to play an important role in determining the antimicrobial activity of the resulting extract [26,51].

Last but not least, although essential oils are Generally Recognized As Safe (GRAS) [22], the effect of high concentrations of these natural compounds on human health raises serious doubts on their effective use in the food industry as natural preservatives. At the same time, many other factors, such as economic costs, legislation, practical effectiveness, and organoleptic effects should be evaluated [49].

## 3. Use of Natural Preservatives to Improve Nutritional and Functional Properties in Cheese

According to the scientific definition, functional foods have been defined as “foods that provide benefit beyond basic nutrition” [61]. Herbs and spices well fit in this statement, because they have numerous bioactive compounds providing potential health benefits [62]. Many scientific works have shown that most of the health effects of herbs and spices on several diseases, such as cancer, cardiovascular disease, arthritis, and mental health protection, may be mediated through their strong antioxidant effects [62]. Therefore, herbs and spices or its extracts can be added to food products as a carrier for nutraceuticals [16].

The antioxidant action of plant-derived compounds is mainly due to the high concentration of phenolic compounds, which have strong H-donating activity [20], but also to other antioxidant compounds, such as carotenoids, phenolic diterpenes, flavonoids, anthocyanidins, etc. [63]. However, while the antioxidant plant activity in vitro is widely demonstrated in the scientific literature, data regarding their in vivo effects are still lacking [20]. In addition, identifying the specific compounds responsible for plant antioxidant activity remains an ongoing issue, and even when focusing on only one phytochemical compound, such as rosmarinic acid (one of the major phenolic compounds in the Lamiaceae family, to which antioxidant activity is attributed), in vitro results do not often agree with in vivo results [64]. Additionally, Bakheit and Foda [65] reported that the antioxidant activity of some spices (black pepper, black cumin, and clove) in vitro, in the powder form, was higher than that of the spicy cheeses.

Cheese also contains low quantity of phenolic compounds, which are retained due to their interaction with milk proteins. However, water soluble compounds with low molecular weight are often lost in the cheese whey [66], so the antioxidant activity of “native” phenolic cheese compounds is very low.

Caleja et al. [58] tried to enrich a cottage cheese with the bioactive compounds of *Foeniculum vulgare* Mill. decoction. In the fennel decoction the authors found 12 phenolic acids and derivatives, and five flavonoids, for a total phenolic compounds of 29.76 mg/g, which resulted in increasing the cheese antioxidant activity up to 14 days of storage.

Solhi et al. [67] improved the functional properties of a processed cheese by adding different amounts of tomato powder: the treated samples showed higher lycopene content than control and, even if lycopene decreased during storage, due to its degradation, at the end of the storage time allowed for processed cheese (two months) the lycopene content of the fortified cheeses was still high. Moreover, tomato powder addition to cheese resulted in higher antioxidant activity of the fortified samples compared to the control.

The antioxidant activity of aqueous extracts of *Inula britannica* flower were evaluated by Lee et al. [68] in a cheddar-type cheese, where cheese fortified with extracts of *Inula britannica* resulted in higher in vivo antioxidant activity. Furthermore, the flavored cheeses showed higher protein, ash, and total phenolic contents compared to the control, together with a decrease in pH, fat, and total solids values [68].

In some cases, it has also been proved that the addition of some spices, such as *Satureja hortenis* L., into cheese not only inhibits the microbial development, but also ensures the intake of some essential elements, such as Fe, of which cheese is lacking [69].

Spearmint essential oil increased the protein content of a white cheese, also during cold storage, while the effects of its addition on the fat and moisture contents of cheese became more evident during storage [70].

Carocho et al. [71] added basil leaves, either in its dehydrated form or as a decoction, in *Serra da Estrela* cheese, observing an increase in the antioxidant activity of cheeses, a decrease in moisture, and a preservation in the unsaturated fatty acid and protein contents. Furthermore, the authors reported higher functionalizing and conservative effect of decoctions compared to the dehydrated form.

## 4. Use of Natural Preservatives to Improve the Sensory Characteristics of Cheese

The use of plants in cheese making is an ancient practice, but it is generally related to local traditions and mainly used to give cheese a particular flavor or aroma or to increase shelf life [72]. 

However, the plant-derived compounds generally have a strong flavor, even when present in small quantities, which could result in a possible consumers’ rejection. Fortunately, the scientific research is currently able to carry out analytical studies on the sensorial properties of cheeses, also due to the use of specialized personnel (panel test), to optimize the use of natural compounds and improve the sensory characteristics of cheese. Gammariello et al. [50], for example, employed a panel test for screening different natural compounds for suitability for dairy applications: the panelists disliked melaleuca and mint smell when applied to Fior di latte, so they discarded them, while thyme, sage, rosemary, sour or sweet orange, vanilla, and grapefruit gained the acceptability from the same panelists.

Another advantage for today’s scientific research is that analytic equipment are also able to evaluate the cheese aroma profile, in order to determine the real contribute of each natural compounds to the cheese aroma. It is well known, in fact, that volatile compounds of essential oils can interact with fat, carbohydrate, and in particular with proteins of the cheese matrix [54,73], thus reducing their ability to be transferred to cheese [73,74]. Moro et al. [49], for example, by using headspace stir bar sorptive extraction coupled to gas chromatography/mass spectrometry, reported a mean total recovery yield of 62.51% for volatile compounds of rosemary essential oil in fortified sheep milk cheeses, with hydrocarbon chemical compounds being transferred in higher amount than oxygenated compounds. The same findings were reported also by Licón et al. [52] when evaluating the carryover effect of different essential oils (from *Melissa officinalis*, *Ocimum basilicum*, and *Thymus vulgaris*) in pressed ewes’ cheese.

Many scientific works are present in the literature concerning the use of natural compounds in food applications [75], and in particular in cheese making, with the aim, among others, at improving cheese sensory characteristics. Plants are generally used in the form of essential oils, in the dried form or after extraction with a solvent (generally water, ethanol, or a mixture of them). Of course, not all attempts have proven successful in improving sensory attributes of cheese.

### 4.1. Essential Oils Affecting Sensonsory Characteristics

The concentration of essential oils (EOs) added to cheese plays a key role in improving its sensory characteristics. Azizkhani et al. [37], in evaluating the inhibitory activities of salvia and basil essential oils in Iranian white cheese, observed significant differences (*p* < 0.05) in odor, color, and texture among the cheeses containing EOs and the control sample. In particular, the cheese containing 0.75% of basil EO gained the highest overall acceptability during storage, followed by sample containing 0.5% of salvia EO, while additions of 0.75% and 1% of salvia EO resulted in cheeses impaired in both odor and taste. Additionally, Abbas et al. [76] observed better sensory attributes in an ultra-filtrated soft cheese when adding low concentrations of basil essential oil compared to the cheeses with a high level of the same natural compound.

Ehsani et al. [38] enhanced all sensory attributes (texture, color, odor, flavor, and general acceptability) of Iranian white cheese by means of black cumin essential oils: all treated samples showed higher scores compared to the control, and cheeses supplemented with lower concentration of black cumin essential oil (1%) was the most preferred by the panel test. These results were in agreement with those reported by Hassanein et al. [29], according to which *Domiati* soft cheeses supplemented with black cumin oil had higher sensory scores than control.

In the study of Laranjo et al. [77], instead, the sensory evaluation reported the rejection of the soft goat cheeses treated with oregano EO and oregano leaves, due to a pronounced bitter taste, even if the cheeses manufactured only with oregano leaves showed high acceptability. In a similar research, Selim [60] observed that a high concentration of clove and tea tree essential oils, necessary to ensure antimicrobial activity, presented a strong off-flavor in *Feta* cheese. Additionally, Foda et al. [70] observed that high concentrations of spearmint essential oil could raise concerns regarding changes in the organoleptic properties of a white cheese, so much so that the panel test showed the highest acceptability at lower concentrations of essential oil. However, the authors also observed that, prolonging the cold storage, the addition of spearmint EO had no significant effect on the cheese organoleptic characteristics. The same phenomenon had been already observed by other authors [78,79].

### 4.2. Dried or Fresh Plants Affecting Sensonsory Characteristics

The addition of fresh or dried herbs has also proven to be a useful tool to improve the sensorial characteristics of cheese, and even in this case the quantity of natural compounds added to food is a key factor in determining the cheese sensorial attributes.

*Satureja hortensis* L. added into a fresh cow cheese significantly improved its smell and taste, with 1% and 1.5% of dried plant showing the highest and lowest score, respectively [69].

Different concentrations of celery leaf in white soft cheese, instead, showed an increase in flavor and overall acceptability, with the highest scores for the addition of 5% and 10% of celery leaves [80]. In a similar research, Al-Obaidi [81] observed no significant differences in color, texture, bitterness and flavor between control cheese and cheeses treated with different concentrations of turmeric powder. However, the cheese treated with the highest concentration of turmeric powder (0.3%, w/v) showed a lower score of flavor than the control. Anyway, turmeric addition resulted in a lower peroxide value during cheese storage, revealing the antioxidant effect on cheese of the phenolic compounds of turmeric powder.

The sensory attributes of a processed cheese were instead enhanced by adding tomato powder to samples [67]: all the fortified cheeses showed higher scores for total acceptance, flavor, and color compared to control, with 2% tomato powder resulting in the highest scores.

Josipović et al. [32] successfully developed thirty types of novel cottage cheeses by adding dried or fresh pepper, parsley, garlic, dill, and rosemary. All fortified cheeses resulted in good sensory properties, with better acceptability of cheeses with fresh pepper or herbs compared to the dry spices. In particular the sample with the highest score (19.50) contained fresh red pepper, while the cheese containing dried parsley got the lowest score (12.11).

Marinho et al. [82] provided evidence that coating of ripened semi-hard caw cheese with lard and dehydrated rosemary leaves improved the physical and physicochemical properties of cheese: the coating, in fact, allowed the final products to retain higher moisture content and preferred texture, appearances, and color. Furthermore, the rosemary gave cheeses a slight aroma besides spicy flavor.

Different scientific works also reported the addition of saffron to cheese [83,84,85] to generally enhance color and flavor of the dairy products [86]. Color and coloring properties of saffron are related to crocins [86], sugar esters of crocetin, while saffron aroma is mainly due to safranal [84], the degradation product of picrocrocin, which is responsible for the bitter taste of saffron [86]. Due to the presence of all these analytes, saffron is also considered a source of bioactive compounds [86]. Significant differences were observed in flavor among control cheese and cheeses added with different saffron concentrations, but as ripening time increased, these differences were less evident [83,85].

### 4.3. Plants Extracts Affecting Sensonsory Characteristics

In a study carried out by Tayel et al. [31] ethanolic cinnamon extract (70%) revealed the most desirable for significant taste and overall quality enhancement of flavored processed cheeses, while lemon grass and cress (*L. sativum*) were the best extracts to improve the cheese odor and color, respectively.

El-Aziz et al. [27] tried to improve the sensory characteristics of an Egyptian soft cheese, made with buffalo milk retentate: soft cheeses flavored with ethanolic ginger extract (70%) became more acceptable, also throughout storage, and showed no molds and yeasts growth during storage, unlike control samples [27].

Additionally, aqueous extracts of pine needles (*Cedrus deodara* (Roxb.) Loud.) used as natural preservatives in low fat *Kalari* cheese showed significantly (*p* < 0.05) higher scores during storage for flavor, texture, and overall acceptability in the treated samples compared to the control [39]. In the same way, aqueous flower extracts of *Inula britannica* increased odor and taste in a *Cheddar*-type cheese [68]. Evstigneeva et al. [87] instead, evaluated the effect of the addition of different concentrations of the aqueous extract of green tea in a cottage cheese: only at levels above 8% tea extracts taste was detected in cheeses, with the levels of 8% and 9% having a pleasant moderately expressed green tea taste and flavor in cottage cheese. In the samples containing tea extracts in the range 10–16% an increase in the bitter taste was observed and correction with taste fillers was required, while cottage cheese with a high level of tea extract (17%) showed tea taste that was too bitter and unpleasant.

Finally, Elsamani et al. [88] developed various cow milk cheeses by adding different concentrations of lupin milk (a water extract from lupin seeds): all fortified cheeses resulted in higher score of flavor compared to control cheese. However, only cheese with low concentrations of lupin milk (25 mL/100 mL of milk) did not show lower overall acceptability compared to control, unlike the high concentrations (50 and 75 mL/100 mL of milk).

An alternative approach in developing new formulation of cheese with natural compounds was that adopted by Fadavi and Beglaryan [89]: the authors, by means of the response surface methodology, investigated the simultaneous effect of different levels of peppermint extract, starter, rennet, and ripening time on the antioxidant activity and sensory score of ultrafiltrated-*Feta* cheese, with the aim of finding the optimum parameters to give the cheese with the highest antioxidant activity and sensory score. The authors’ results showed that the rennet concentration and the ripening time had, respectively, a negative and positive effect on the antioxidant activity. Peppermint extract, instead, played a crucial role in the acceptance of the cheese samples and showed a negative effect on the sensory score. Therefore the authors proposed the following best solution: 227 μg/g cheese for peppermint extract, 2.7 g/100 kg retentate for starter, 1.3 g/100 kg retentate for rennet, and 41.7 days for ripening time, which resulted in the highest consumers acceptability (sensory score 5.02) and the highest functional value of the cheese (antioxidant activity 48%).

## 5. Use of Natural Preservatives to Extend Cheese Shelf Life

The food quality is generally susceptible to changes during storage due to exposure to heat, enzymes, transition metal ions, oxygen, and light, resulting in potential food degradation or formation of active flavor compounds [90]. In particular dairy products, as well as other water–oil emulsions, can suffer hydrolytic and oxidative rancidity [91], resulting in the release of some volatile fatty acids (C4–C10), their subsequent conversion to other acids and/or ethyl esters, oxidation of lipids to form secondary oxidation products, essentially aldehydes, and production of some organic acids [90]. Most of these compounds are responsible of the bed odor and taste of cheese.

Extending the cheese shelf-life is an important factor in the dairy industry because it can decrease the economic impact, by reducing losses attributed to spoilage, and can allow the cheese accessibility to new and more distant markets [60]. Jalilzadeh et al. [92] reviewed the recent techniques carried out to increase the shelf-life of cheeses, such as the addition of preservatives to modified atmosphere packaging, the active coatings with antimicrobial agents, and the edible packaging based on proteins, polysaccharides, and lipids with various functional additives. However, little attention has been paid on the treatment of cheeses with natural preservatives.

Addition of preservatives is one of the simplest and oldest ways to prolong the cheese shelf life, due to their effect on delaying alterations caused by microorganism growth or keeping the physical properties, chemical composition, and original nutritional value of cheese unchanged during storage [92]. Alternative preservation techniques using naturally derived ingredients are being investigated in cheese making, with the use of spices and herbs being attracting even more interest.

Asensio et al. [90] evaluated different Argentinean oregano EOs as natural preservatives in organic cottage cheese. No effect of the oregano EOs on the cheese ripening was observed, but they helped in the preservation of the flavored cheeses by decreasing the formation of organic acids like lactic, formic, and acetic acids, as well as by lowering the rate of lipid oxidation indicators (hydroperoxide values and conjugated dienes) and the degradation of some unsaturated fatty acids (linolenic, elaidic, and linoleic acids).

Makhal et al. [93] evaluated the improving in the shelf life of direct acidified cottage cheese, one of the cheese suffering of a limited shelf life (general about 7 days) due to its high moisture content (75%) and relatively high pH (5.0). The authors showed that an addition of 40 ppm 30% thymol solution in butter oil enhanced the cottage cheese shelf life by 8 days compared to the control sample, without having noticeable adverse effect on the typical flavor of cottage cheese, unlike 50 ppm 30% thymol solution.

Mohamed et al. [51] succeeded in extending the shelf life of a cream cheese up to four weeks by adding *Moringa oleifera* extract.

Recent developments have also been done in the field of cheese packaging, where the application of antimicrobial agents to packaging materials could be useful to prevent the growth of microorganisms on the surface and hence leading to an extension of the food shelf-life [94]. Tsiraki and Savvaidis [95], for example, through microbiological, physical, chemical, and sensory analyses, demonstrated how basil essential oil, in addition to modified atmosphere packaging or under vacuum, can prolong the shelf life of a Greek whey cheese about 10–12 days and 6 days, respectively. Conte et al. [96], instead, evaluated the effectiveness of different antimicrobial packaging systems on the bacterial growth during the storage of *Mozzarella*: a lemon extract, at three different concentrations, was used as an active agent, in combination with the brine and with a gel solution based on sodium alginate. The authors demonstrated that, even in conditions of “thermal abuse” (15 °C), the mozzarella shelf life is longer. The addition (1–3%) of *Pimpinella saxifraga* essential oil in sodium alginate coating of a fresh cheese, instead, extended the shelf life of cheese up to 10 days, by decreasing the lipid oxidation and improving microbiological stability during storage [97].

Although the encouraging results, more studies are needed to confirm the effectiveness of natural preservatives in extending the cheese shelf-life: in fact, there is a great variability in the types of cheese on the market (fresh, ripened, pasta filata, or blue cheeses) and different natural preservatives could have dissimilar behavior in these type of cheeses.

## 6. Plant-Based Preservatives in Cheese Making: the Present and the Future

In a time where consumers are more aware and interested in what they eat, the use of natural preservatives has been becoming a trend that is followed by food manufacturers. This has led the scientific research to be increasingly addressed towards the evaluation of plant essential oils and plant polyphenols as natural antimicrobials in foods [33,98]. These plant-based compounds also offer the advantage of being rich in bioactive molecules and having strong antioxidant activity [15].

Herbs and spices have found many applications in the dairy sector as antioxidant, antimicrobial, and flavoring ingredients, as well as to improve the appearance and attractiveness of fortified foods for consumers and to increase the sale of the vegetable products [16].

Although quite promising, more research is needed to evaluate the real use of these plant-derived ingredients in the food industry, and in particular in cheese making processes. There are many in vitro studies, but in vivo research are still lacking [20]. The food matrix interaction with the antimicrobial mechanism of the natural compounds, which can result in a decreased antimicrobial effect, has yet to be well understood. Furthermore, the real effectiveness of these natural preservatives depends on the quantity added to food, but the potential adverse effects of the natural compounds on the sensory characteristics of the cheese are not always taken into account. Not the least, economic and regulator aspects: the price of natural preservatives must be reasonable when compared with the synthetic compounds carrying out the same effect, otherwise they will not be considered by the food industry, and the approval by the governmental authorities is needed, but legislation on natural additives is still limited and often confusing [15].

Another aspect that the authors want to focus on, and that, in our opinion, has not been taken into account yet by the scientific research, is the potential use of natural preservatives to further improve the nutritional value in cheese. Herbs and spices, in fact, can be used in different recipes to partially or wholly replace less desirable ingredients, such as salt, sugar, and added saturated fat [62]. Therefore, they could play an important role in the dairy sector as partial or full substitutes of salt, one of the main ingredients in many cheeses. Salt, in fact, is a fundamental factor in cheese making because it adds flavor to cheese, helps to dry the curd, is essential in the development of a good rind, and exerts antimicrobial activity when used as a brine or as dry salting. According to the European Food Safety Authority EFSA database on food consumption, milk, and dairy products are among the most consumed foods by adults in Europe, showing that the average consumption of cheese for a European adult is 34.2 g/day [99]. A negative aspect related to the cheese consumption is the resulting average daily intake of salt. Reducing the salt content to less than 5 g/day, according to the WHO recommendations [100], represents an important challenge to reduce the development of diseases, such as hypertension and cardiovascular diseases, related to excessive salt consumption. Even if many attempts have been reported in the literature aiming at reducing the salt content in cheese making [101,102,103], at our knowledge no research has been carried out to evaluate the partial or full substitution of salt with natural preservatives, such as herbs and spices. Therefore, also the research in this area should be encouraged.
